# Holocarboxylase Synthetase Deficiency: A Second Case Report With Neonatal Cholestatic Liver Disease

**DOI:** 10.1002/jmd2.70051

**Published:** 2025-12-10

**Authors:** Sophie Manoy, Claire Murray, Matthew Lynch, Tahlee Minto, Kelvin Choo, Carolyn Bursle, Michelle Lipke, Jim McGill, Anita Inwood, David Coman

**Affiliations:** ^1^ Department of Metabolic Medicine Queensland Children's Hospital Brisbane Australia; ^2^ School of Medicine University of Queensland Brisbane Australia; ^3^ Department of Paediatric Surgery Queensland Children's Hospital Brisbane Australia; ^4^ Department of Chemical Pathology Royal Women's and Brisbane Hospital Brisbane Australia; ^5^ School of Nursing and Social Work University of Queensland Brisbane Australia; ^6^ School of Medicine Griffith University Gold Coast Australia

**Keywords:** biotin, holocarboxylase synthetase deficiency, inborn error of metabolism, liver disease, metabolic acidosis

## Abstract

Holocarboxylase synthetase deficiency is an autosomal recessive inborn error of metabolism characterised by life‐threatening metabolic acidosis, ketoacidosis and hyperammonaemia through reduced biotin‐dependent carboxylase activity. We report the presentation of a Polynesian neonate with severe metabolic acidosis secondary to holocarboxylase synthetase deficiency with the development of cholestatic liver disease thought to be secondary to holocarboxylase synthetase deficiency. This is only the second reported case of holocarboxylase synthetase deficiency associated with cholestatic liver disease. Both of these cases were a result of the same homozygous c.647T>G L216R pathogenic variants in the *HLCS* gene suggesting a possible genotype–phenotype correlation and broadening the phenotypic understanding of this disease.

## Key Points

- We describe the second reported case of holocarboxylase synthetase deficiency secondary to homozygous c.647T>G L216R variants associated with cholestatic liver disease.
- This strengthens understanding of a genotype–phenotype correlation in holocarboxylase synthetase deficiency and broadens the phenotypic understanding of this disease.
- The patient responded well to biotin therapy.

## 1 Introduction

Holocarboxylase synthetase deficiency (HLCSD) is an autosomal recessive condition caused by biallelic pathogenic variants in the *HLCS* gene (*HLCS*, OMIM 609018), resulting in reduced or absent activity of holocarboxylase synthetase [1]. Biotin, an essential B vitamin, acts as a coenzyme to five carboxylases (acetyl CoA carboxylase 1, acetyl CoA carboxylase 2, pyruvate carboxylase, 3‐methylcrotonyl CoA carboxylase and propionyl CoA carboxylase) [2]. Holocarboxylase synthetase catalyses the covalent binding of biotin to inactive forms [3, 4]. Inherited deficiency of this enzyme causes decreased carboxylase activity, resulting in HLCSD (HLCSD, OMIM 253270) [5]. As carboxylases play an essential role in the catabolism of amino acids, gluconeogenesis and fatty acid synthesis, their deficiency causes life‐threatening metabolic derangements [6].

HLCSD is characterised by metabolic acidosis, ketoacidosis and hyperammonaemia, and typically presents in the neonatal period, although late‐onset presentations have been reported [7, 8, 9]. The clinical presentation is similar to that of other organic acidurias [10] with the addition of cutaneous symptoms such as rash [11].

HLCSD is fatal if untreated. Pharmacological doses of free biotin can reverse both clinical and biochemical features [12, 13], however outcomes are varied with intellectual disability and episodes of profound metabolic acidosis described. There have been reports of partial or very limited responses to biotin therapy depending on the genotype [13, 14, 15, 16] with this case's homozygous c.647T>G L216R variants previously reported to result in minimal responsiveness [15].

The diagnosis of HLCSD is suggested by a characteristic urine organic acid profile demonstrating elevated lactate, 3‐hydroxyisovalerate, 3‐hydroxypropionate, 3‐methylcrotonylglycine, methylcitrate and tiglylglycine. Confirmation is via enzyme assay demonstrating reduced HLCS activity, or DNA sequencing identifying biallelic pathogenic variants in the *HLCS* gene. Antenatal diagnosis and successful treatment with maternal biotin administration have also been reported previously [17, 18].

We report the case of a Polynesian neonate presenting with HLCSD and cholestatic liver disease. Cholestasis relating to giant cell hepatitis has been reported only once in HLCSD in the literature; this case broadens the possible phenotypic understanding of the disease [19].

## 2 Case Report

A female infant was born at term gestation via emergency caesarean section for fetal distress. She was the fourth child to non‐consanguineous parents of Cook Islander and Samoan background. There was no significant family history. An ultrasound scan at 33 weeks gestation was abnormal with the identification of prominent cerebral ventricles. A fetal brain MRI showed subependymal cysts with evidence of associated haemorrhage. A chromosomal microarray performed antenatally via chorionic villus sampling showed no copy number abnormalities but demonstrated long contiguous stretches of homozygosity.

Meconium‐stained liquor was present at delivery and the baby was born in poor condition, requiring intubation and cardiac chest compressions. The initial examination was unremarkable for the localisation of pathology. Venous blood gas showed a high anion gap metabolic acidosis with an elevated lactate (see Table 1), persistent despite fluid replacement and sodium bicarbonate correction. The ammonia level was elevated at 160 μmol/L (< 50 μmol/L). The possibility of an inborn error of metabolism such as an organic aciduria was considered based on these findings. Serial venous blood gas results are shown in Table 1.

**TABLE 1:** Serial venous blood gas results.

Age	pH (7.32–7.43)	pCO_2_ (mmHg) (38–54)	HCO_3_ (mmol/L) (18–27)	BE (mmol/L) (−2.0 to 3.0)	Lactate (mmol/L) (0.5–2.2)
1 h	7.14	33	11	−18	12.7
8 h	7.16	8	3	−26	17.8
12 h	7.12	11	4	−25.7	19.0
14 h	7.01	15	4	−27	17.0

Further investigations showed a complex urinary organic acid pattern and abnormal newborn screen dried blood spot acylcarnitine analysis. This showed an elevation in 3‐hydroxyisovalerate and propionylcarnitine, consistent with HLCSD. A plasma acylcarnitine profile demonstrated elevations in propionylcarnitine and 3‐hydroxyisovalerylcarnitine, supporting the diagnosis. An MRI brain showed connatal cysts and delayed myelination with a resolving area of right sided haemorrhage. An echocardiogram showed a structurally normal heart. A multiple carboxylase deficiency gene panel later revealed homozygous c.647T>G p.Leu216Arg variants in the *HLCS* gene classified as pathogenic.

Haemofiltration was commenced for the management of persistent metabolic and lactic acidosis with hyperammonaemia. Intravenous 10% dextrose fluids and parenteral nutrition were used. Peak lactate levels were 25 mmol/L (0.5–2.2 mmol/L). Oral biotin and intravenous levocarnitine were commenced. On day two of life the lactate remained elevated at 17 mmol/L, and an abdominal x‐ray revealed necrotising enterocolitis (NEC), which was managed medically. The lactate normalised by Day Four of life. Haemofiltration was ceased and the patient was commenced on enteral feeds with improvement in acidosis and ammonia level. She was discharged home on oral biotin.

On Day 19 of life the patient presented with abdominal distension, poor feeding and vomiting. She was jaundiced. Investigation revealed cholestatic liver dysfunction with conjugated hyperbilirubinaemia. There was no impairment in hepatic synthetic function. These results are shown in Table 2.

**TABLE 2:** Liver function test results.

Age	Bilirubin (total) (μmol/L) (< 20)	Bilirubin (conjugated) (μmol/L) (< 10)	Gamma‐GT (U/L) (15–132)	Alanine transaminase (U/L) (< 25)	Aspartate transaminase (U/L) (< 49)
Day 19	164	77	219	38	68
Day 20	130	76	141	89	158

An emergency laparotomy for bowel obstruction revealed a stricture at the junction of the descending and sigmoid colon with a contained perforation, consistent with late effects of the recent episode of NEC. The distal colon was divided and a colostomy was formed. An intraoperative cholangiogram was normal. A liver biopsy showed minor mixed inflammatory cell infiltrate including lymphocytes, eosinophils and occasional neutrophils without interface activity or piecemeal necrosis. There was no evidence of ductal injury. The lobules showed mild canalicular cholestasis with occasional bile plugs and intact lobular architecture. These features were consistent with a mild cholestatic pattern of injury suggestive of giant cell hepatitis.

The cholestatic liver function derangement persisted and the patient was commenced on ursodeoxycholic acid. Investigations for other causes of severe cholestatic liver disease including infection, alpha 1 antitrypsin deficiency, metabolic liver disease, cystic fibrosis, biliary atresia and structural abnormalities of the biliary tree were negative.

The large bowel stoma was reversed at 3 months of age, by which time the liver function abnormalities had also resolved spontaneously. At six months the patient continued on regular biotin. She had adequate growth on standard infant formula and was transitioning to solids with an individualised low protein feeding plan. She had a maximum fasting time of 6 h. She is globally developmentally delayed, unable to sit unsupported and babbling for vocalisation. However, she is gaining skills over time. On examination she is developing spasticity, predominantly involving her hands for which she is receiving therapeutic intervention.

## 3 Discussion

HLCSD has been reported with an increased incidence in the Polynesian population. The homozygous c.647T>G L216R variant seen in this case represents a founder mutation and is associated with a severe form of the disease [14, 19]. Individuals homozygous for this mutation have been considered largely unresponsive to biotin treatment with a higher mortality [2], although recent reports have suggested more positive outcomes when treated with high doses above 10–20 mg/kg [19]. Our patient continues on oral biotin 60 mg/day (20 mg/kg/day) and has had minimal episodes of metabolic decompensation.

Case reports suggest a broad spectrum of developmental outcomes in HCLSD, ranging from normal growth and development in children who are well controlled, to severe and progressive disability in children with incomplete response to biotin [5, 14, 19]. The developmental progress and emerging neurological signs in our patient might be explained by early brain injury secondary to metabolic decompensation during her initial presentation or could be reflective of her underlying disease process.

HLCSD was suspected in our patient based on her clinical presentation in the context of antenatal imaging findings and her Polynesian ethnicity. The family had been reviewed in the antenatal period for abnormal features on fetal brain MRI. Although a chromosomal microarray and perinatal infectious screening were performed at the time, further definitive diagnostic investigations were not performed. Bandaralage et al. reported common imaging findings in HLCSD in a review of 75 reported patients [20]. Subependymal cysts and ventriculomegaly were seen in 85% of those imaged. Intraventricular haemorrhage and intrauterine growth restriction were also seen. They concluded that HLCSD should be considered in children of Polynesian ethnicity and suggestive antenatal imaging findings [20].

Our patient developed cholestatic liver dysfunction within the second week of life, despite treatment with biotin and subsequent resolution of other biochemical abnormalities. This did not resolve until 3 months of age and extensive investigations including liver biopsy did not reveal an underlying aetiology. Cholestatic jaundice with associated liver function derangement has been previously reported in a Polynesian infant presenting with HLCSD secondary to homozygous c.647T>G L216R variants [19]. Other possible causes of cholestatic liver disease in this case include intestinal obstruction, exposure to antibiotics and prolonged fasting. Cholestatic liver disease is seen in other inborn errors of metabolism including alpha 1 anti‐trypsin deficiency, Niemann‐Pick type C and peroxisomal biogenesis disorders [21]. In this instance the cholestatic picture was thought to be secondary to giant cell hepatitis, a nonspecific histological pattern identified in neonatal liver disease of various aetiologies. Our patient did not have other significant findings on liver biopsy and with no other aetiology found on extensive investigation the liver dysfunction was thought to be related to her underlying HLCSD. This is the second report of cholestatic liver dysfunction secondary to HLCSD.

## 4 Conclusion

We describe the presentation of HLCSD in a Polynesian neonate secondary to homozygous c.647T>G L216R variants with characteristic neuroimaging findings. The patient responded well to biotin therapy. Her clinical course was complicated by cholestatic liver dysfunction which was thought to be secondary to her underlying disorder. This is the second report of cholestatic liver dysfunction secondary to HLCSD with the same genotype, possibly broadening the phenotype of this disease and strengthening understanding of a genotype–phenotype correlation.

## Author Contributions

S.M., C.M. and D.C. prepared the original manuscript. All authors provided input into the manuscript. D.C. provided oversight. All authors take responsibility for the content of the article.

## Funding

The authors have nothing to report.

## Ethics Statement

Local human research ethics committee protocols were followed including obtaining informed consent with a caregiver.

## Consent

Informed consent for preparation of this case report was obtained with a caregiver.

## Conflicts of Interest

The authors declare no conflicts of interest.

## Data Availability

The data that support the findings of this study are available on request from the corresponding author. The data are not publicly available due to privacy or ethical restrictions.
